# ﻿A new species of *Rhopalopsole* (Insecta, Plecoptera, Leuctridae) from the Anhui Province of China and phylogeny of Leuctridae based on mitochondrial genomes

**DOI:** 10.3897/zookeys.1244.137740

**Published:** 2025-07-08

**Authors:** Xiao Yang, Yu-Zhou Du

**Affiliations:** 1 School of Plant Protection & Institute of Applied Entomology, Yangzhou University, Yangzhou 225009, China Yangzhou University Yangzhou China; 2 Jiangsu Province Engineering Research Center of Green Pesticides, Yangzhou University,Yangzhou 225009, China Yangzhou University Yangzhou China

**Keywords:** Geographical distribution, phylogeny, *Rhopalopsolegibba*, stonefly, taxonomy

## Abstract

A new species of Leuctridae (Plecoptera) from Anhui Province of China, *Rhopalopsolegibba***sp. nov.**, is described and illustrated. The new species is compared to a similar species, *R.dicondylica* Yang & Du, 2021. We sequenced the complete mitochondrial genome of the new species and found a length of 16,157 bp. Phylogenetic analysis based on 10 complete or nearly complete mitogenomes of four genera of Leuctridae from China showed the following phylogenetic relationships: ((*Rhopalopsole* + *Perlomyia*) + *Paraleuctra*) + *Leuctra*. Finally, the geographical distribution of Leuctridae in China is preliminarily summarized.

## ﻿Introduction

*Rhopalopsole* Klapálek, 1912 is a species-rich genus in the family Leuctridae, with more than 80 valid species known from the Oriental and eastern Palaearctic regions (Klapálek 1912; Okamoto 1922; Wu 1949, 1973; Jewett 1958, 1975; Illies 1966; Kawai 1968, 1969; Harper 1977; Zwick 1977; Harrison and Stark 2008; Sivec et al. 2008; Stark and Sivec 2008; Stark et al. 2012; Qian 2012; Yang et al. 2015; DeWalt et al. 2024). Currently, more than 50 species of this genus have been recorded from China, with recent contributions made by Yang et al. (2004, 2006, 2009), Sivec et al. (2008), Li and Yang (2010, 2011, 2012), Li and Yang. (2010, 2011), Qian and Du (2011, 2012a, b, 2013, 2017), Chen and Du (2017), Mo et al. (2018), Yang and Du (2021a, b, c, 2022), Yang et al. (2022), Li et al. (2017), Chen et al. (2022), Li et al. (2022), Liu et al. (2023), Li et al. (2023), and Yang and Du (2024a, b, 2025a).

Anhui Province, located on the eastern border of China, is a transitional region between the Palaearctic and Oriental regions. The unique geographical location and favourable climatic conditions provide a good living environment for leuctrids.

Recently, we examined material of Plecoptera from Anhui Province and found a new species of *Rhopalopsole* Klapálek, 1912. Moreover, we sequenced the complete mitochondrial genome of the new species. Because the phylogenetic position of genera of Leuctridae is unclear, we constructed and studied phylogenetic trees of four genera in China. These results provide a basis for further phylogenetic studies of Leuctridae worldwide. Finally, the geographical distribution of Leuctridae in China is preliminarily summarized.

## ﻿Materials and methods

Specimens were collected by hand and preserved in 75% ethanol. Morphological details were examined with a Leica MZAPO microscope. Colour illustrations were taken with a Keyence VHX-5000. All specimens used in this study are deposited in the Insect Collection of Yangzhou University (**ICYZU**), Jiangsu Province, China. The morphological terminology follows that of Sivec et al. (2008).

All specimens were kept in anhydrous ethanol at −20 °C. The genomic DNA was extracted from the thoracic muscles and legs of adults using the column mtDNAoutKit (Axygen Biotechnology, Hangzhou, China), following the manufacturer’s instructions, and stored at −20 °C until used for PCR.

In this paper, MitoZ v. 2.4 was used to sequence assemble and annotate the clean data (Meng et al. 2019).

A phylogenetic analysis was carried out based on the complete or nearly complete mitogenomes of 10 species of Leuctridae. One species (*Taeniopteryxugola*) from Taeniopterygidae was selected as an outgroup (Table 1). Each PCG and rRNA was individually aligned using the MAFFT algorithm (Katoh and Standley 2013). Three datasets were concatenated for phylogenetic analyses: PCG matrix, including 13 complete PCGs; PCG12 matrix, including the first and second codon positions of the thirteen PCGs; and PCGR matrix, including thirteen complete PCGs and two rRNA.

**Table 1.** Taxonomic and molecular information and accession numbers of species analyzed in this study.

Family	Species	Genbank ID	Length	AT%
Taeniopterygidae	*Taeniopteryxugola*	NC_037897.1	15353	69.8
Leuctridae	*Leuctrafusca*	MT872701.1	15342	66.4
	*Leuctrahippopus*	MT483625.1	16070	67.6
	*Paraleuctracercia*	NC_053557.1	15625	67.4
	*Paraleuctraorientalis*	PQ360799	15617	67.3
	*Perlomyiaisobeae*	NC_053558.1	15795	71.8
	*Perlomyialevanidovae*	OQ612624.1	15774	71.5
	*Perlomyiakappa*	OQ612623.1	15759	72
	*Rhopalopsolebulbifera*	NC_042207.1	15599	70.7
	*Rhopalopsolegibba*	PQ360798	16157	68.6
	*Rhopalopsolesubnigra*	OQ612622.1	15562	69.7

Maximum- likelihood phylogenies were inferred using IQ-TREE v. 1.6.12 (Nguyen et al. 2015) under the Edge-linked partition model for 1000 ultrafast (Minh et al. 2013) bootstraps, as well as the Shimodaira–Hasegawa-like approximate likelihood-ratio test (Guindon et al. 2010). Bayesian inference phylogenies were inferred using MrBayes v. 3.2.7a (Ronquist et al. 2012) under a partition model (2 parallel runs, 10 million generations), in which the initial 25% of sampled data were discarded as burn-in.

The best partition scheme and model of nucleotide sequences for the ML and BI methods were selected by ModelFinder (Kalyaanamoorthy et al. 2017).

## ﻿Results and discussion

### ﻿Taxonomy

#### ﻿ Rhopalopsole gibba

Taxon classificationAnimaliaPlecopteraLeuctridae

Yang & Du sp. nov.

D67F8139-48BF-5743-B2B5-BB11BAAF26F6

https://zoobank.org/B015CBF3-BF3D-4548-BDBA-03965A86970A

##### Description.

**Figure 1.**
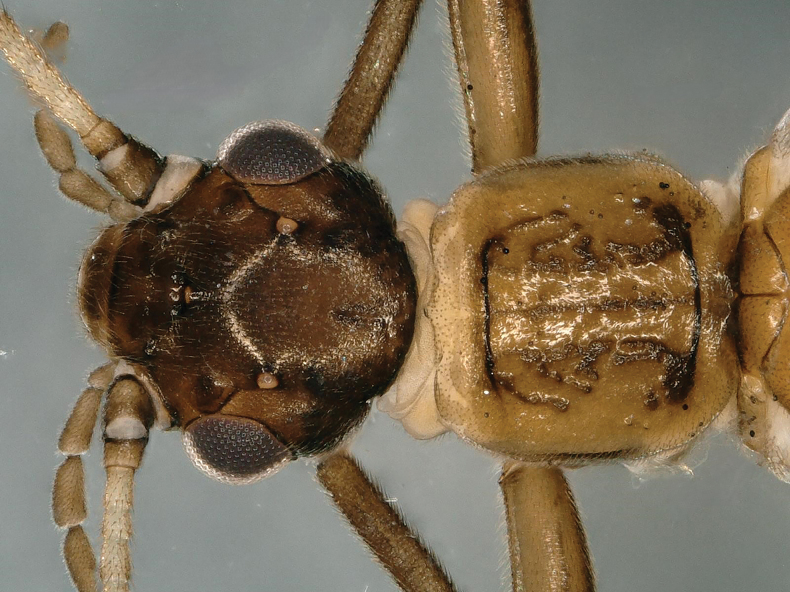
*Rhopalopsolegibba* sp. nov. Male head and pronotum, dorsal view.

**Male.** Body length 5.7–5.9 mm. Forewing length 5.4–5.5 mm, hindwing length 4.2–4.3 mm. Head dark brown, wider than pronotum; ocelli pale brown; antennae and palpi light brown. Pronotum brown, quadrate. Legs brown. Wings hyaline and veins light brown (Fig. 1).

**Figure 2.**
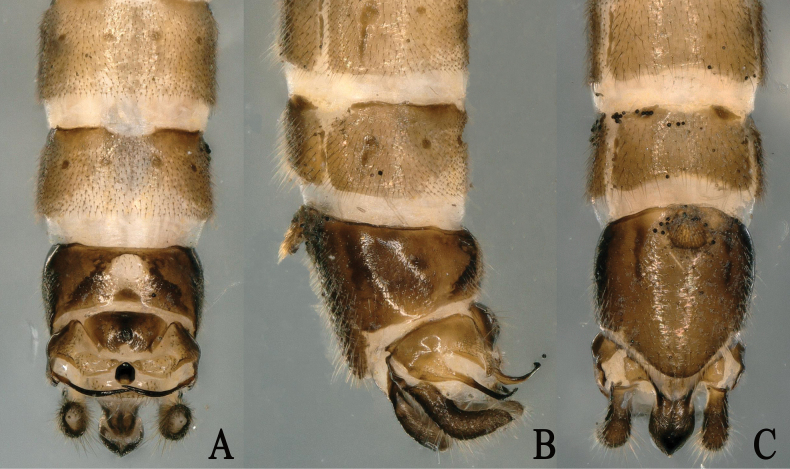
*Rhopalopsolegibba* sp. nov. **A.** Male terminalia, dorsal view; **B.** Male terminalia, lateral view; **C.** Male terminalia, ventral view.

Tergum 9 mostly sclerotized, somewhat less so on median circular area with two strong sclerotized marks. Sternum 9 basally with subcircular vesicle bearing dense hairs, apically with a comparatively longer subgenital plate. Tergum 10 bearing a large central plate covered laterally with macrotrichia and a mammillary process on both sides; middle strongly sclerotized part of the central plate with small round field of knobs. Transverse plates nearly triangular and less hairy with some seta. Lateral projections of tergum 10 long and sinuous. Epiproct thick at base, not tapering appreciably along its length ending in a downcast point. Subanal lobes large, narrow at base expanding distally, then narrowing to end. Cercus hairy and upcurved, with a small spine (Fig. 2).

**Female.** Unknown.

**Egg and nymph.** Unknown.

##### Type materials.

**Figure 3.**
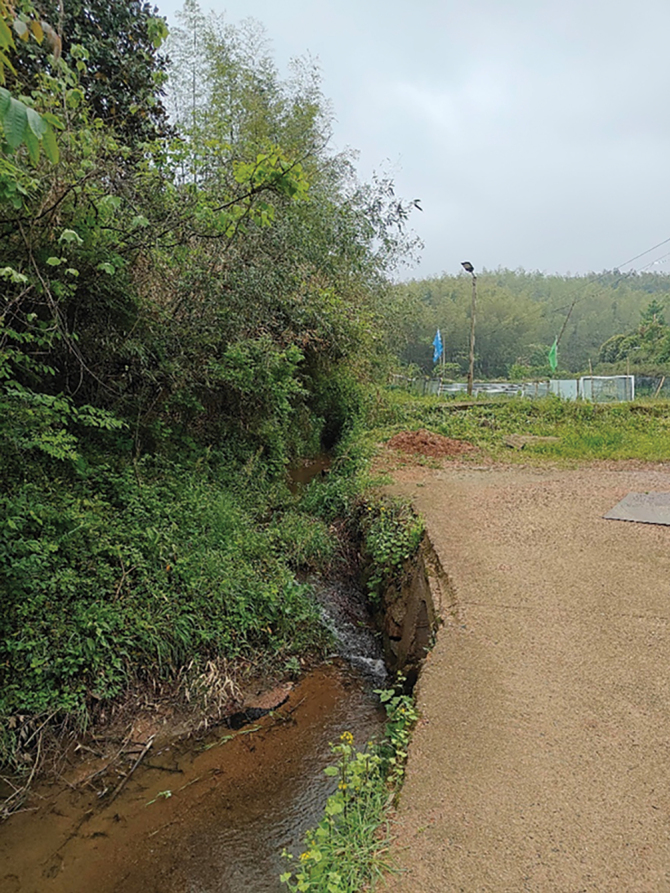
Type locality of *Rhopalopsolegibba*: a stream in Yaocun town, Anhui Province, China (photo provided by Huo Qing-Bo).

***Holotype***: • ♂, China: Anhui Province, Xuancheng City, Yaocun town, alt. 726 m, 30°53'24"N, 119°6'30"E, 2024-VI-13, leg. Huo Qing-bo and Zeng Liang-liang (Fig. 3). ***Paratypes***: • 4 ♂♂, same data as holotype.

##### Etymology.

The species name refers to the central plate with a small round field of gibba at the middle of the tergum 10.

##### Diagnosis and remarks.

The new species, *Rhopalopsolegibba*, is similar to members of the *R.dentata* group (Sivec et al. 2008), particularly *Rhopalopsoledicondylica* Yang & Du, 2021. The males share the similar shape of epiproct and the shape of the subanal lobes. In *R.gibba*, the cercus is hairy and upcurved, with a small spine and tergum 10 bears a large central plate covered laterally with macrotrichia and there is a mammillary process on both sides; the middle strongly sclerotized part of the central plate with small round field of knobs. However, in *R.dicondylica*, the cerci lack spines and the hind border of tergum 10 bears a large central plate covered laterally with microtrichia, and there is a mammillary process on both sides; the middle poorly sclerotized part of the central plate is triangular and rounded apically (Fig. 2).

### ﻿Phylogenetic analyses

Few studies have examined phylogenetic relationships among Leutridae genera. South et al. (2021) used transcriptome data for a 2021 study of plecoptera phylogeny in North America. However, his study did not include *Rhopalopsole* data, so the incidence of Chinese Leutridae could not be determined. Data on the genus *Leuctra* were also lacking in the studies by Guo et al. (2022), Rehman et al. (2023) and Cao et al. (2024).

**Figure 4.**
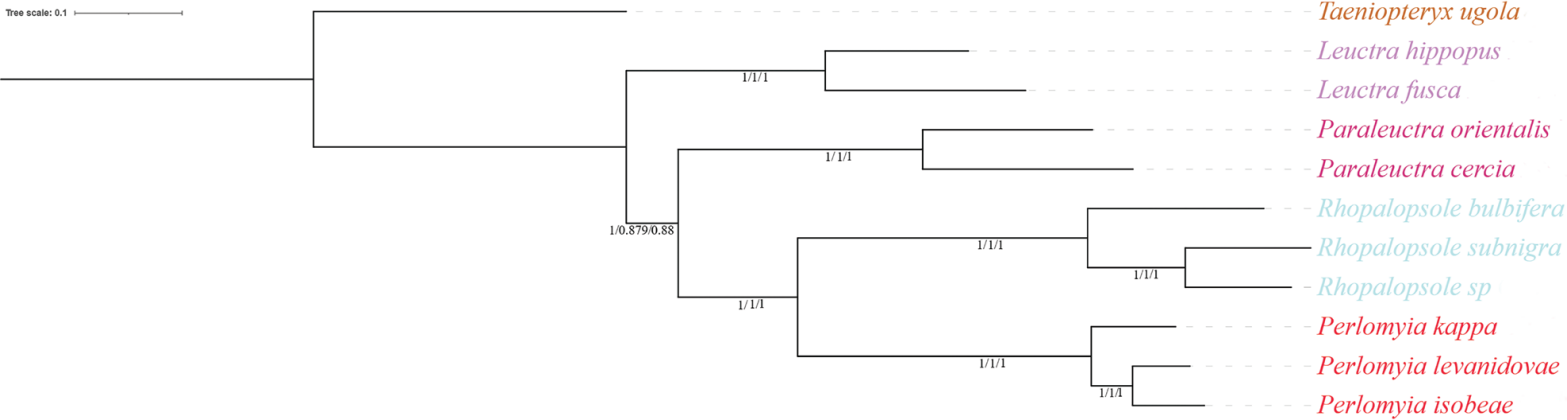
Phylogenetic trees obtained from the Bayesian inference and maximum-likelihood analyses. The congruent topology from the analysis of BI–PCG (PP in left), BI–PCG12 (PP in middle), and BI–PCGR (PP in right). Note: PP, posterior probability.

**Figure 5.**
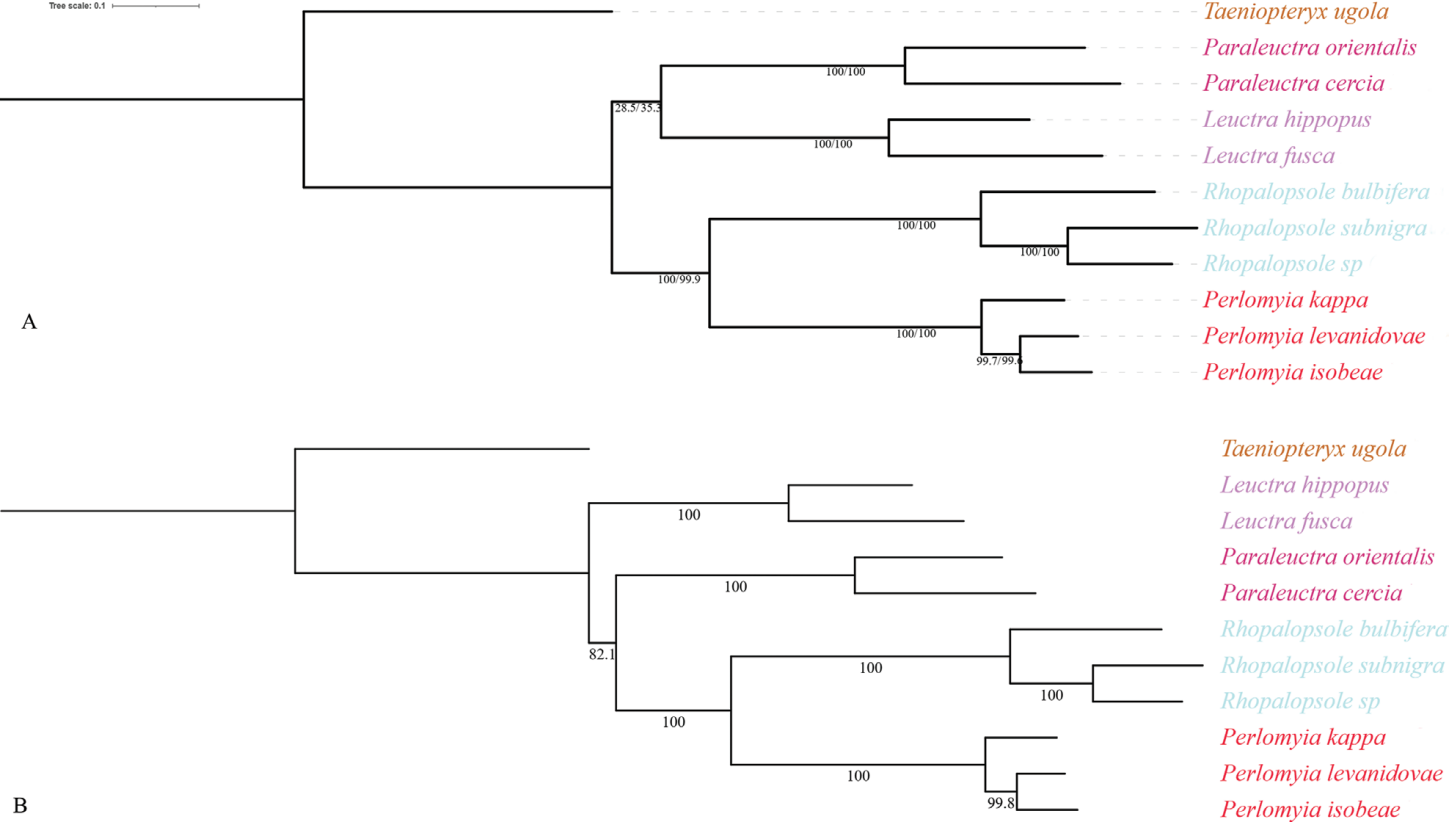
Phylogenetic trees obtained from the maximum-likelihood analyses. **A.** The congruent topology from the analysis of ML–PCG (BP in left), and ML–PCG12 (BP in right); **B.** The congruent topology from the analysis of ML–PCGR. Note: PP, posterior probability; BP, bootstrap value.

In this study, six phylogenetic trees were generated from three matrices using both ML and BI methods. The ML and BI analyses based on the PCG and PCGR matrix generated phylogenetic trees with the same topologies and high nodal supports (Figs 4, 5). But in the PCG12 matrix, ML and BI analyses produce different topologies. The result generated by BI is consistent with the topology generated by PCGR, which is ((*Rhopalopsole* + *Perlomyia*) + *Paraleuctra*) + *Leuctra* (Figs 4, 5). The ML analyses based on PCG and PCG12 matrix relationships of Leutridae are as follows: (*Leuctra* + *Paraleuctra*) + (*Perlomyia* + *Rhopalopsole*) (Fig. 5).

Although the ML tree results based on the PCG and PCG12 matrices are different, considering the relative support values for certain nodes, we consider that the former topology as best reflecting the phylogeny of Leutridae of China, viz.: ((*Rhopalopsole* + *Perlomyia*) + *Paraleuctra*) + *Leuctra*.

### ﻿Distribution of *Leuctridae* in China

So far, 70 species of Leuctridae insects in four genera have been recorded in China. The distribution of each genus was obtained from the analysis of known species (Table 2). As can be seen in Table 2, most species of Leuctridae have little or no distribution in the Qingzang, Mengxin, and Northeast regions because the most widespread *Rhopalopsole* is not adapted to the climate in these areas. The Northeast region, as well as Central, South, and Southwest China, mostly belong to the temperate zone and subtropical monsoon climate, and the climate is suitable for leuctrids. In addition, species of leuctrids in China are basically endemic to China, and only nine species are distributed abroad (such as Russia and Vietnam). This is a phenomenon of species dispersion, but the range of dispersion is limited to the Palaearctic and Oriental Realms, and there are no species that cross to other realms.

**Table 2.** Distribution of Leutridae by genus and species in China.

Species	SCR	CCR	SR	NCR	QR	MR	NR	CES
*Leuctrafusca*		√	√	√	√	√	√	
*L.cercia*							√	
*Paraleuctracervicornis*			√					√
*Par.cuihuashana*				√				√
*Par.orientalis*		√	√	√				
*Par.qilianshana*						√		
*Par.zapekinae*							√	√
*Perlomyiaangulata*	√							√
*Per.excavata*	√							√
*Per.levanidovae*							√	
*Per.smithae*							√	
*Per.taiwanensis*	√							√
*Rhopalopsoleampulla*		√						√
*R.apicispina*		√		√				√
*R.baishanzuensis*		√						√
*R. ﻿basinigra*	√	√	√	√				√
*R.bawanglinga*	√							√
*R.bispina*		√	√	√				√
*R.brevicula*	√							√
*R.brevidigitata*	√							√
*R.cestroidea*	√							√
*R.curvispina*		√						√
*R.dentata*	√							
*R.dentiloba*	√							√
*R.dicondylica*	√							√
*R.emeishan*		√	√	√				√
*R.exiguspina*		√						√
*R.faciursina*	√							√
*R.fengyangshanensis*		√						√
*R.flata*	√	√						√
*R.furcospina*	√	√						√
*R.guangdongensis*	√							√
*R.gutianensis*	√	√						√
*R.hainana*	√							√
*R.hamata*		√						√
*R.horvati*			√	√		√		√
*R.intonsa*		√						√
*R.jizushana*	√							√
*R.lii*			√					√
*R.liui*		√						√
*R.longispina*	√	√						√
*R.longtana*		√						√
*R.magnispina*			√					√
*R.meilan*	√							√
*R.memorabilis*		√						√
*R.minutospina*		√						√
*R.nanlinga*	√							√
*R.pseudodentata*	√							
*R.qinlinga*				√				√
*R.recurvispina*		√		√				√
*R.shaanxiensis*				√				√
*R.shimentaiensis*	√							√
*R.siculiformis*	√							√
*R.sinensis*	√	√	√	√				
*R.singiplatta*			√					√
*R.spiniplatta*		√						√
*R.subnigra*	√							√
*R.taiwanica*	√							√
*R.tianmuana*		√						√
*R.triangulis*	√							√
*R.trichotoma*		√						√
*R.tricuspis*				√				√
*R.triseriata*			√					√
*R.wolong*					√			√
*R.wuyishanensis*		√						√
*R.wuzhishana*	√							√
*R.xui*	√	√						√
*R.yadonga*					√			√
*R.yunnana*			√					√
*R.zhejiangensis*		√						√

Note: South China Region, SCR; Central China Region, CCR; Southwest Region, SR; North China Region, NCR; Qingzang Region, QR; Mengxin Region, MR; Northeast Region, NR; Chinese Endemic Species, CES.

The distribution of these four genera in China also provides supporting evidence for our phylogenetic relationship results. China has a vast territory, spanning the Palaearctic and Oriental Realms, and due to the lack of obvious geographical barriers and barriers between the two realms, a transition zone is formed, which allows the species of the two realms to mix with each other. China’s animal biogeographical regions are divided into two realms, three sub-realms, and seven regions. These are the South China, Central China, Southwest, North China, Qingzang, Mengxin, and Northeast regions. Among these, the Northeast, North China, Mengxin, and Qingzang regions belong to the Palaearctic Realm, while the Southwest, Central China, and South China regions belong to the Oriental Realm.

Among the 13 genera of Leuctridae, *Leuctra* has the largest number of species and the most widespread distribution, covering the Holarctic Realm. In China, there is only one widely distributed species, *Leuctrafusca* (L.), found in provinces such as Qinghai, Sichuan, Guizhou, Ningxia, Jilin, Zhejiang, etc. We suggest that it might be distributed in other provinces as well, but it may not have been detected due to incomplete collecting. It belongs to the transition area between the Oriental-Nearctic and tends toward a distribution in the Nearctic Realm.

*Leuctra* and *Paraleuctra* are distributed widely. *Perlomyia* is only found in the Northeast region and Taiwan Province. *Rhopalopsole* insects are one of the most widely distributed leuctrids in China. They are mostly distributed in the Oriental region and the transitional region between the Oriental region and the Palaearctic region. Therefore, we conclude that the widely distributed *Leuctra* have formed population differentiation under different geographical conditions. The divergent evolution of different geographic populations will be the focus of further research.

## Supplementary Material

XML Treatment for Rhopalopsole gibba

## ﻿Additional information

### Use of AI

No use of AI was reported.

### Author ORCIDs

Xiao Yang https://orcid.org/0000-0001-6829-8542

Yu-Zhou Du https://orcid.org/0000-0002-4452-7125

### Data availability

All of the data that support the findings of this study are available in the main text or Supplementary Information.

## Supplementary materials

Supplementary material 1 Best scheme and model for this phylogeny study Data type xlsx https://binary.pensoft.net/file/1369865This dataset is made available under the Open Database License (http://opendatacommons.org/licenses/odbl/1.0/). The Open Database License (ODbL) is a license agreement intended to allow users to freely share, modify, and use this Dataset while maintaining this same freedom for others, provided that the original source and author(s) are credited.Xiao Yang, Yu-Zhou Du
